# No evidence for widespread positive selection on double substitutions within codons in primates and yeasts

**DOI:** 10.3389/fgene.2022.991249

**Published:** 2022-09-09

**Authors:** Frida Belinky, Anastassia Bykova, Vyacheslav Yurchenko, Igor B. Rogozin

**Affiliations:** ^1^ National Center for Biotechnology Information, National Library of Medicine, National Institutes of Health, Bethesda, MD, United States; ^2^ Life Science Research Centre, Faculty of Science, University of Ostrava, Ostrava, Czech Republic

**Keywords:** natural selection, tandem mutations, short-term evolution, neutral evolution, double substitutions, positive selection, negative selection, purifying selection

## Abstract

Nucleotide substitutions in protein-coding genes can be divided into synonymous (S) and non-synonymous (N) ones that alter amino acids (including nonsense mutations causing stop codons). The S substitutions are expected to have little effect on function. The N substitutions almost always are affected by strong purifying selection that eliminates them from evolving populations. However, additional mutations of nearby bases can modulate the deleterious effect of single N substitutions and, thus, could be subjected to the positive selection. This effect has been demonstrated for mutations in the serine codons, stop codons and double N substitutions in prokaryotes. In all abovementioned cases, a novel technique was applied that allows elucidating the effects of selection on double substitutions considering mutational biases. Here, we applied the same technique to study double N substitutions in eukaryotic lineages of primates and yeast. We identified markedly fewer cases of purifying selection relative to prokaryotes and no evidence of codon double substitutions under positive selection. This is consistent with previous studies of serine codons in primates and yeast. In general, the obtained results strongly suggest that there are major differences between studied pro- and eukaryotes; double substitutions in primates and yeasts largely reflect mutational biases and are not hallmarks of selection. This is especially important in the context of detection of positive selection in codons because it has been suggested that multiple mutations in codons cause false inferences of lineage-specific site positive selection. It is likely that this concern is applicable to previously studied prokaryotes but not to primates and yeasts where markedly fewer double substitutions are affected by positive selection.

## Introduction

In classic population genetics, co-localized substitutions are assumed to occur one at a time, independently of one another. However, clustering of mutations, in particular, those occurring in adjacent sites (multiple nucleotide mutations) has been documented in many diverse organisms (Averof et al., 2000; Drake et al., 2005; Drake, 2007; Schrider et al., 2011; Stone et al., 2012; Terekhanova et al., 2013; Harris and Nielsen, 2014; Besenbacher et al., 2016). Double substitutions within the same codon in protein-coding genes have also been claimed to be driven by positive selection. This conclusion stemmed from comparisons of the observed frequencies of double substitutions to those expected from the frequencies of single substitutions: if the frequency of a double substitution is significantly greater than the product of the frequencies of the respective single substitutions, positive selection is inferred (Bazykin et al., 2004; Rogozin et al., 2016; Belinky et al., 2018). This observation is consistent with the possibility that a prevalence of positively selected double nucleotide mutations is compensation for the first deleterious mutation through subsequent positive selection acting on the second substitution (Bazykin et al., 2004; Rogozin et al., 2016; Belinky et al., 2018). Positive selection affecting double substitutions has been detected as a general trend in the rodent lineage (Bazykin et al., 2004). Similarly, signatures of positive selection have been found for double substitutions in stop codons in prokaryotes (UAG → UGA and UGA → UAG), which could be attributed to the deleterious non-stop intermediate, UGG (Belinky et al., 2018) and double substitutions in two disjoint series of codons for serine (Rogozin et al., 2016). Thus, multiple nucleotide mutations in codons potentially could originate from selection, mutational biases including clusters of mutations (Averof et al., 2000; Drake et al., 2005; Drake, 2007; Schrider et al., 2011; Stone et al., 2012; Terekhanova et al., 2013; Harris and Nielsen, 2014; Besenbacher et al., 2016) or a combination of both these factors.

Previously, we assessed the selection that affects double substitutions within codon in prokaryotes (Belinky et al., 2019). Briefly, we compared the frequency of each such double substitution to the frequency of a double synonymous substitution in adjacent codons with the same base composition (Belinky et al., 2019). Although it is well known that transition (A:T ↔ G:A) and transversion (A:T ↔ T:A, A:T ↔ C:G, G:C ↔ C:G) rates differ substantially, the differences between different combinations of specific transitions and transversions are less thoroughly characterized, and it is not clear to what extent adjacency of mutations is modulated by base composition. We thus compared all codon double substitutions to their respective double synonymous substitutions with the same nucleotide changes. In many cases, it was found that a codon double substitution has a significantly higher double/single ratio, compared to the same double synonymous substitution, suggesting that these are true cases of positive selection that acts on the second substitution and brings it to fixation in prokaryotes (Belinky et al., 2019).

In this paper the same methodology was applied for analyses of selection in yeasts and primates (including human). No signs of wide-spread positive selection were detected. This result suggests major differences in selection modes between prokaryotes (Belinky et al., 2019) and two studied eukaryotic lineages (primates and yeasts). This is likely to be important for inference of lineage-specific site positive selection.

## Materials and methods

### Datasets

**FIGURE 1:**
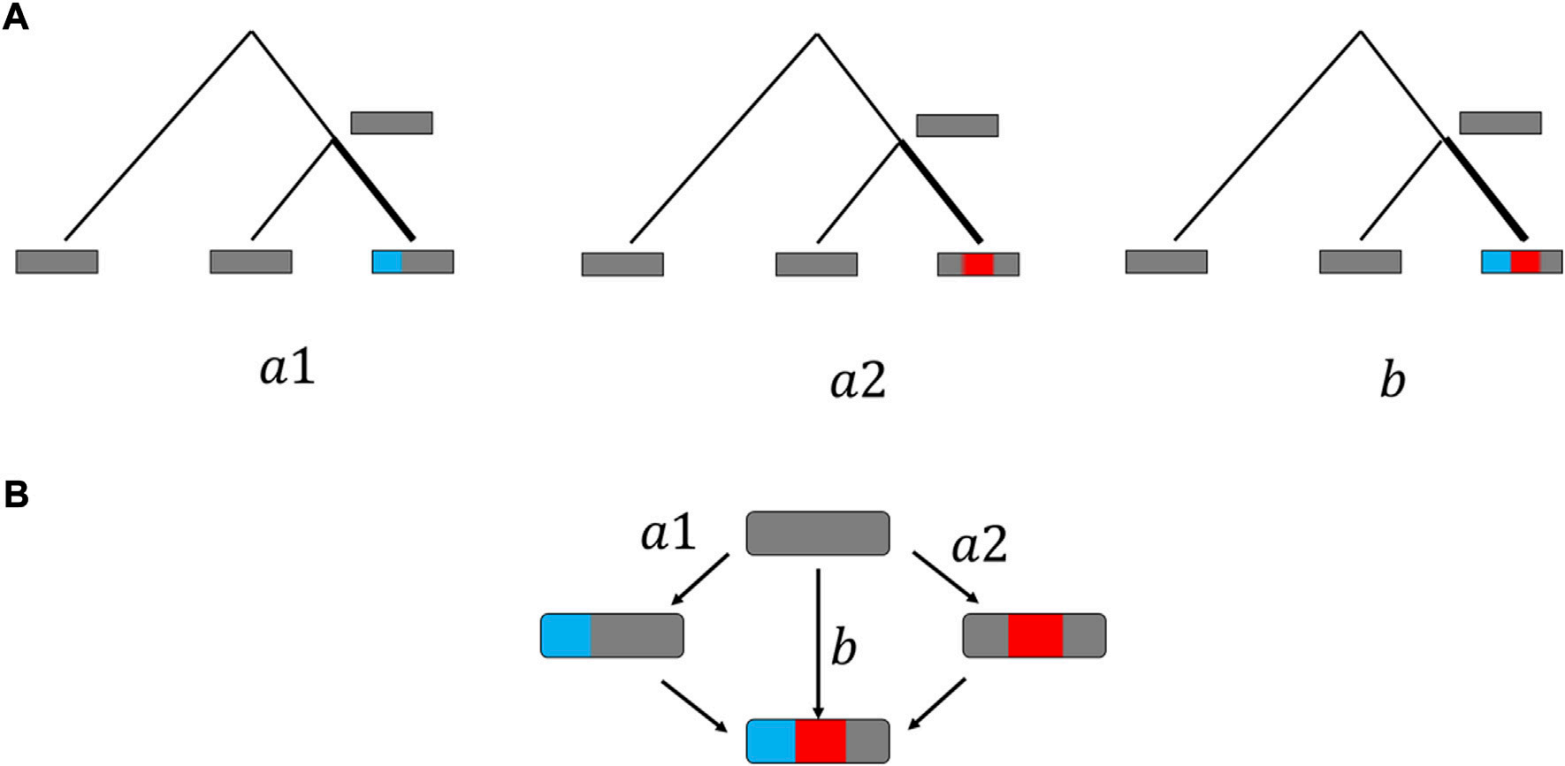
Conceptual scheme of double substitution analysis. **(A)**Single or double substitutions are inferred from the genomic data by construction of genomes triplets and relying on parsimony principle (see Material and Methods). **(B)** Point mutations are assumed to appear one at a time, such that observed double substitutions **(B)** occur through intermediate single substitutions states. For each double substitution, there are two possible single substitution pathways **(a1, a2)**. The double fraction DF is calculated as the ratio between the number of double substitutions **(b)** and the sum of relevant single (a1+a2) and double **(b)** substitutions.

To reconstruct mutations in protein-coding DNA under the parsimony principle, we inferred and analyzed single and double substitutions in triplets of closely related primates and yeasts as previously described (Rogozin et al., 2016). In brief, the parsimony principle implies that mutations occur along the thick branches in the trees (Figure 1A) assuming that there is no mutation or one mutation per each position. Whole-genome alignments of three yeast species (*Saccharomyces cerevisiae*, *S. paradoxus*, and *S. mikatae*) were downloaded from the *Saccharomyces* Genome Database (SGD, www.yeastgenome.org/). Local alignments of protein-coding regions were extracted using the SGD orthology assignments (Rogozin et al., 2016). Protein-coding sequences for primates (*Homo sapiens*, *Callithrix jacchus* and *Otolemur garnettiiwere*) and their orthology assignments were obtained from Ensembl databases as previously described (Belinky et al., 2018). Briefly, protein-coding sequences were downloaded for each species from the Ensembl database, as well as orthology assignments from Ensembl mart (Kersey et al., 2016). Genes with ‘one-to-one’ orthology were aligned using MAFFT with the -linsi algorithm (Katoh et al., 2005). In total, 15,234 primate and 4,100 yeast gene alignments were used for further analyses.

### Analysis of codon double substitutions

Details of analyses of double substitutions in codons are described in (Belinky et al., 2019). Here, we provide a brief description of the methodology. For each codon change (Figure 1B), the frequency of change to any other codon was the number of changes divided by the number of ancestral reconstructions of this codon based on the parsimony principal. For each double substitution the double/single ratio was the observed double substitution frequency divided by the cumulative single substitution frequency. For example, for the change AAA→GGA the double/single ratio was the observed frequency of AAA→GGA divided by the cumulative counts of AAA→AGA, AAA→GAA and AAA→GGA. Thus, for each double substitution (Figure 1) the following data were collected and estimated:

1) The double substitution count (b in the Figure 1).
2) The single substitution count (which is the summation of the two single counts (a1 and a2 in the Figure 1).

We used double fractions (DFs) as a measure of selection. The DF is calculated as the observed double substitution count (b in the Figure 1) divided by the sum of the single (a1 and a2 in the Figure 1) and double substitution counts:

DF=b/(a1+a2+b)

The selection on double substitutions was analyzed by comparing DF for within-codon double substitutions to two null models described below.

### Analysis of double synonymous substitutions in adjacent codons—null models

**FIGURE 2:**
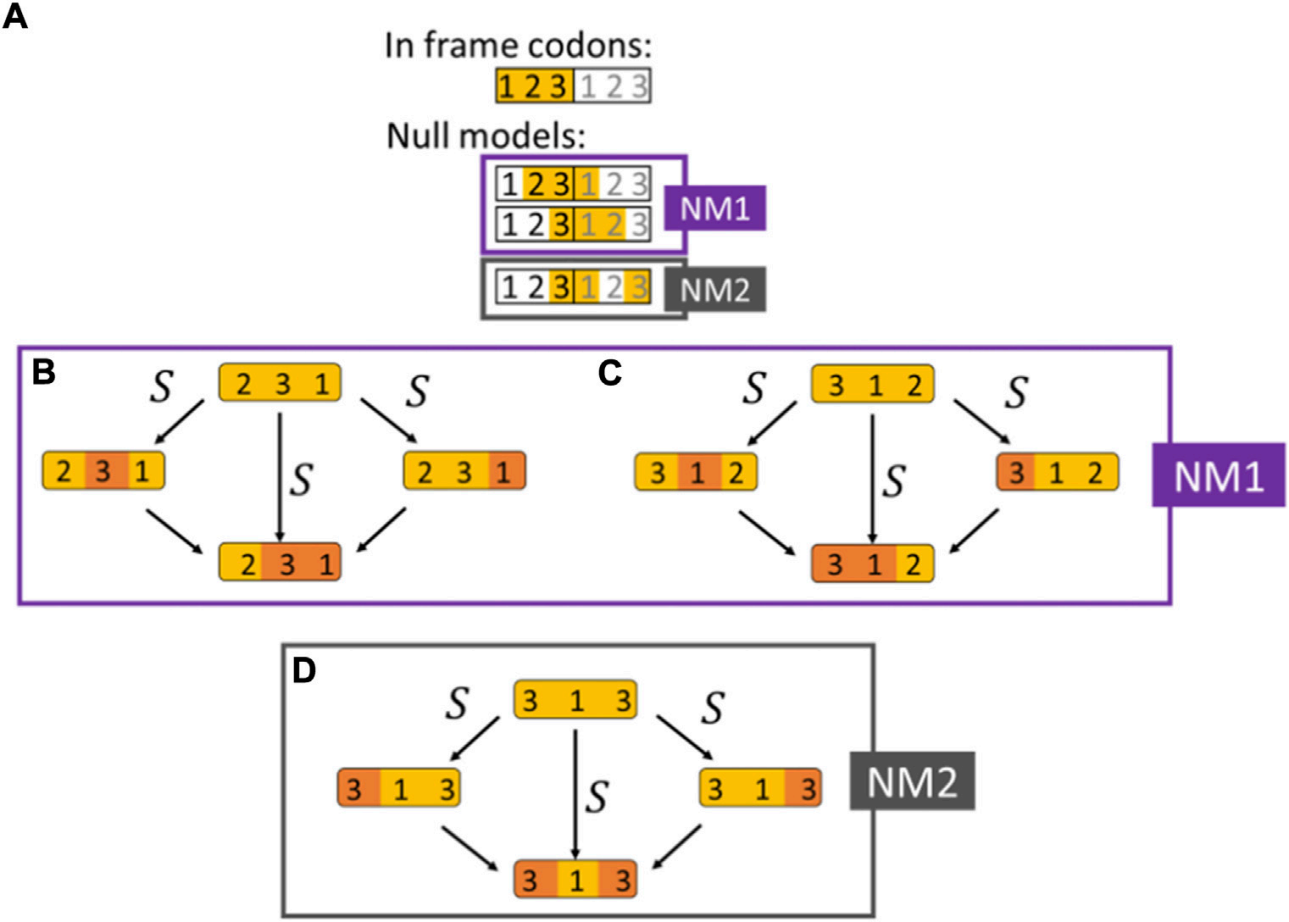
Double synonymous substitutions in adjacent codons used as null models. **(A)** The selection on double substitutions inferred by comparing the DF for codons and their respective null models shown in orange (NM1 and NM2). Two adjacent codons are illustrated, and the nucleotide position within the codon is indicated according to the reading frame. The three null models are artificial codons constructed by considering positions from two adjacent codons. **(B)** Null model NM1 (the 321 configuration). An invariant 2nd codon positions in the first codon, followed by a 4-fold degenerate site in the 3rd positions of the first codon, that is, followed by a 2-fold degenerate site in the 1st codon position of the 2nd codon. **(C)** Null model NM1 (the 312 configuration). A 4-fold degenerate site in the 3rd codon position followed by a 2-fold degenerate site in the 1st codon position of the second codon, that is, followed by an invariant base in the 2nd codon position of the second codon. **(D)** Null model NM2. A 4-fold degenerate site in the 3rd position of the 1st codon followed by an invariant 1st position in the second codon and by a 4-fold degenerate site in the 3rd codon position (skipping the 2nd position of the 2nd codon).

For double synonymous substitutions in adjacent codons, we collected the same data as for codon double substitutions in codon-like 3-base sequences with three possible configurations (Figure 2):

A. An invariant 2nd codon positions followed by a 4-fold degenerate site in the 3rd codon positions, that is, followed by a 2-fold degenerate site in the 1st codon position of the next codon (the 231 configuration, Figure 2B).

B. A 4-fold degenerate site in the 3rd codon positions, that is, followed by a 2-fold degenerate site in the 1st codon position of the next codon, that is, followed by an invariant base in the 2nd codon position of the second codon (the 312 configuration, Figure 2C).

C. A 4-fold degenerate site in the 3rd codon positions, that is, followed by an invariant 1st codon position in the second codon of which the 2nd position is disregarded and followed by a 4-fold degenerate site in the 3rd codon position (Figure 2D).

The first codon in configurations A-B can be any of the 4-fold degenerate codons, i.e, codons for L, V, S, P Y, A, R and G, and the second codon of configurations A-B can be either a codon for R or L which are the only two amino acids that have a degenerate 1st codon position. An additional restriction for configurations A-B is that the ancestral state of the 3rd codon position of the 2nd codon is a purine (A/G) since only then the 1st codon substitution can be synonymous. Similarly, the 1st and 2nd codons configuration C can be any of the 4-fold degenerate codons.

### Assignment of codon double substitution types

**FIGURE 3:**
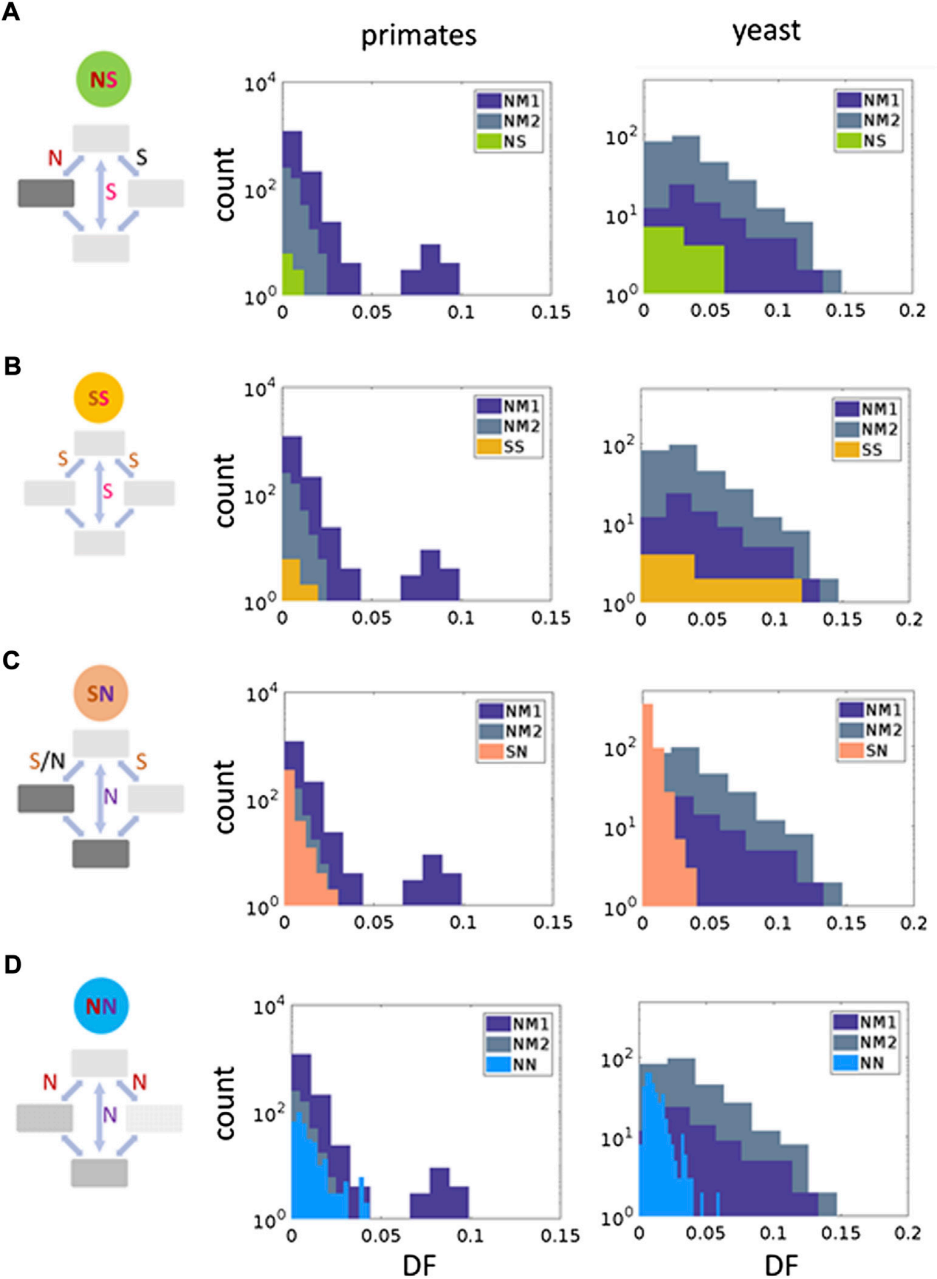
**S**elective regimes of the codon double substitutions in primates and yeasts. Right panels show a classification of codon double substitution based on the synonymy of the ancestral vs. the final codon state, and the synonymy of the ancestral to the intermediate codons. Two left panels show comparisons of DF for each codon double substitution class to the double synonymous null models (NM1 and NM2) using the Mann–Whitney U test. **(A)** NS, one non-synonymous intermediate, synonymous final codon. Primates: NM1 *p*-value = 0.77, NM2 *p*-value = 0.72. Yeasts: NM1 *p*-value = 0.06, NM2 *p*-value = 0.25. **(B)** SS, double synonymous codon substitutions. Primates: NM1 *p*-value = 0.42, NM2 *p*-value = 0.17. Yeasts: NM1 *p*-value = 0.82, NM2 *p*-value = 0.45. **(C)** SN, at least one synonymous intermediate codon, non-synonymous final codon. Primates: NM1 *p*-value = 2.38 × 10^−63^, NM2 *p*-value = 8.73 × 10^−34^. Yeasts: NM1 *p*-value = 4.28 × 10^−38^, NM2 *p*-value = 5.64 × 10^−98^. **(D)** NN—both intermediates and the final codon are non-synonymous to the ancestral. Primates: NM1 *p*-value = 0.059, NM2 *p*-value = 2.53 × 10^−5^. Yeasts: NM1 *p*-value = 7.16 × 10^−27^, NM2 *p*-value = 5.61 × 10^−56^.

For each codon double substitution there are two distinct paths to get from the ancestral state codon to the final (derived) codon state, with each step in the path having a single substitution to reach an intermediate state codon (Figure 2). Each step can be either synonymous or non-synonymous, and the ancestral vs. final codon could be either non-synonymous or synonymous. Some codon substitution could have a stop as an intermediate codon in one of the paths, these cases were disregarded in the current analysis. In this analysis we assigned the combination type to each codon double substitution based on the synonymy of the ancestral to the intermediate codons, and the synonymy of the ancestral vs. the final codon state (Figure 3, left panels and Supplementary Figure S2). NS denotes codon double substitutions in which (at least) one of the intermediates is non-synonymous while the final codon is synonymous compared to the ancestral codon (Figure 3A and Supplementary Figures S1D S2). SS denotes codon double substitutions in which both intermediates and the final codon are all synonymous codons (Figure 3B and Supplementary Figure S2). SN denotes codon double substitutions in which (at least) one intermediate is synonymous while the final codon is nonsynonymous compared to the ancestral codon (Figure 3 and Supplementary Figure S2). NN denotes codon double substitutions in which both intermediates are nonsynonymous, and the final codon is also nonsynonymous compared to the ancestral one (Figure 3D and Supplementary Figure S2).

### Statistical testing

Fisher’s exact test was used to compare the number of double codon substitutions to single cumulative substitutions, to test for significant differences in DF between codon double substitutions and the comparable null models. An example of the comparison of the non-adjacent codon double substitution CTT→TTA is shown in the Supplementary Figure S1D. The Mann–Whitney U test was used to compare the DF values between each of the codon double substitution types (SS, SN, NS, NN) and each of the null models (NM1 and NM2). The Bonferroni correction was applied to correct for multiple testing.

## Results

### Different types of codon double substitutions in primates and yeasts

Representing all within-codon double substitutions in the general form, “ancestral-intermediate-final”, we define the following 4 combinations of codons: 1) SS is “S intermediate—S final” codons, 2) SN is “S intermediate—N final” codons, 3) NS is “N intermediate—S final” codons, 4) NN is “N intermediate—N final” codons (Figure 3, left panels and Supplementary Figure S2) (Rogozin et al., 2016; Belinky et al., 2018; Belinky et al., 2019).

Similar to our previous study of double substitutions in prokaryotes (Belinky et al., 2019), we consider three types of codon-like double synonymous substitutions that were used as null models for the double substitutions in codons (Supplementary Figure S1). The selection pressure on each codon double substitution is assessed by comparing the double/single substitution ratio DF (that is, the ratio of the frequency of a double substitution to the sum of the frequencies of the single and double substitutions in the respective codon positions) to that for double synonymous substitutions (Supplementary Figure S1). The DF is assumed to be mostly affected by the substitution rate at the second step (from intermediate codons to final codons, Figure 1B). Thus, a significantly lower DF compared to that of the corresponding double synonymous substitution will be indicative of purifying selection, and conversely, a higher ratio will point to positive selection.

Comparisons of double mutation DF values with null models NM1 and NM2 (Figure 3, central and right panels) suggested that the dominant mode of selection is purifying selection. In all eight studied cases in primates and yeasts the mean DF values is smaller than DF values for null models (Figure 3). These differences are statistically significant for NN and SN values (Figure 3). The NM1 model tends to produce wider distributions compared to NM2 model (Figure 3). This is likely to be due to a higher frequency of tandem mutations compared to mutations separated by one nucleotide (Averof et al., 2000; Drake et al., 2005; Drake, 2007; Schrider et al., 2011; Stone et al., 2012; Terekhanova et al., 2013; Harris and Nielsen, 2014; Besenbacher et al., 2016).

### Modes of selection in specific codon double substitution classes in primates

**FIGURE 4:**
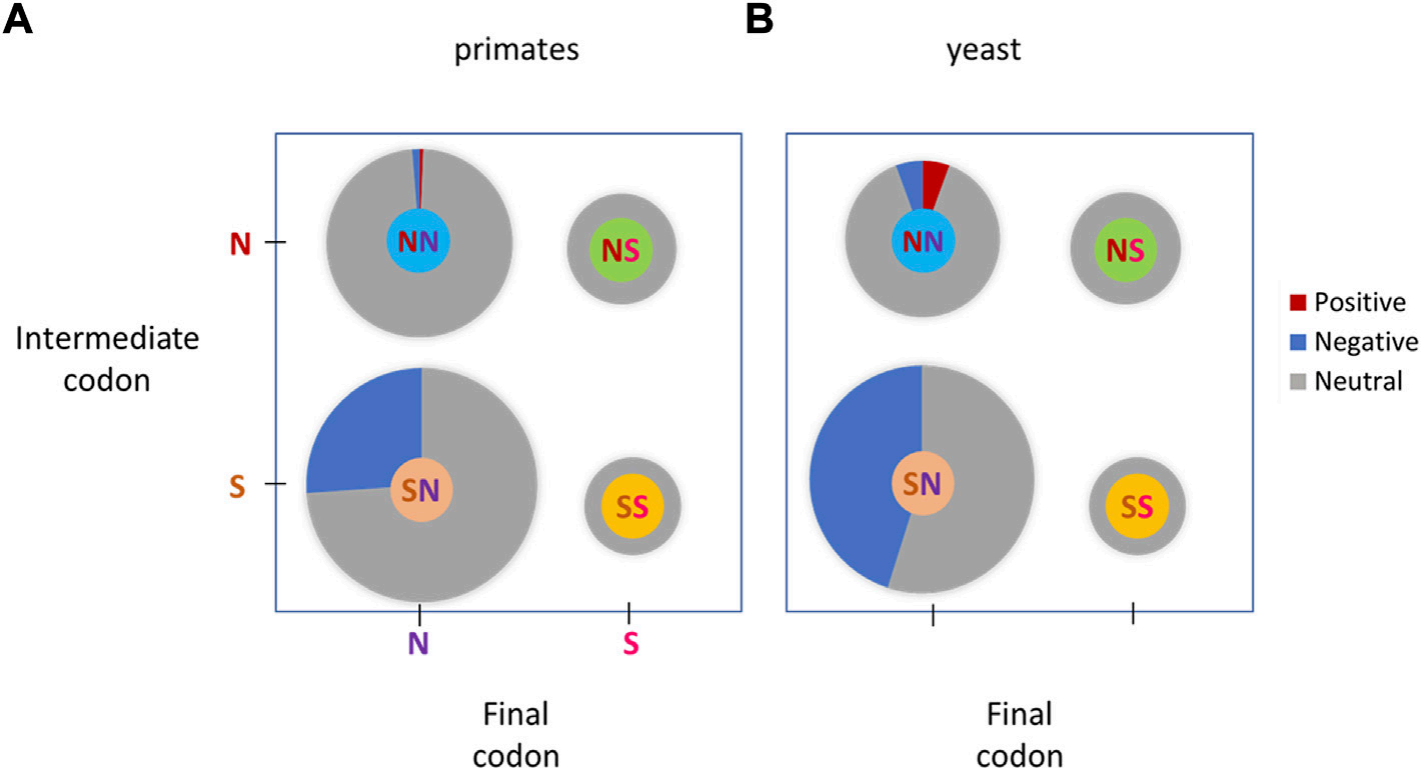
Selective pressure in different codon double substitutions classes. Positive, combinations compatible with positive selection, where a codon double substitution has a significantly higher DF than the corresponding DF of a null model (NM1 or NM2). Negative, combinations compatible with purifying selection, where a codon double substitution has a significantly lower DF than the corresponding DF of a null model. Neutral, combinations, compatible with neutral evolution, where the codon DF was not significantly different from that of the corresponding DF of a null model. **(A)**, primates; **(B)**, yeasts.

We analyzed four types of double substitutions in more detail. To characterize the modes of selection that affect each codon double substitution in greater detail, the frequency of each codon double substitution was compared to the same codon-like substitution pattern in a double synonymous null model (Figure 2). Each codon double substitution is compared to either NM1 or NM2 depending on the distance between the substituted bases (Supplementary Table S1). In total, of the 716 codon double substitutions compared (Supplementary Table S1), only <1% (2 cases after Bonferroni correction) had significantly higher DF compared to the equivalent double synonymous substitutions (Supplementary Table S1), which is compatible with positive selection, and 15% (104 cases after the Bonferroni correction) had significantly lower DF, compatible with purifying selection (Figure 4A and Supplementary Table S1). This result suggests that positive selection affects a negligible fraction of double substitutions in codons although these cases may be false positives. A substantial fraction of double substitutions is subject to purifying selection (Figure 4A).

For NS and SS double substitutions no signs of positive or negative selection were detected (Figure 4A). A significant trend of purifying selection on codon double substitutions is evident in combination SN (Figure 4A), in which double substitutions have significantly lower DF compared to the double synonymous DF (Figure 4A). Combination NN (312 instances) has 2 cases with codon under positive selection and 4 cases compatible with purifying selection, thus neutrality cannot be rejected for the entire group (Figure 4A). The individual cases in combination NN that are compatible with positive selection are TTT → GGT (F → G) and TTT → GCT (F → A) (Supplementary Table S1).

### Modes of selection in specific codon double substitution classes in yeasts

Highly similar results were obtained for yeasts (Figure 4B). In total, of the 317 codon double substitutions compared (Supplementary File S1), only 1% (4 cases after Bonferroni correction) had significantly higher DF compared to the equivalent double synonymous substitutions (Supplementary Table S1), which is compatible with positive selection. This result suggests that positive selection affects a negligible fraction of double substitutions in codons although these cases may be false positives. 34% of studied (108 cases after the Bonferroni correction) had significantly lower DF, which is compatible with purifying selection. A substantial fraction of double substitutions is likely to be subject to purifying selection (Figure 4B).

For NS and SS double substitutions no signs of positive or negative selection were detected (Figure 4B). A significant trend of purifying selection on codon double substitutions is evident in combination SN (Figure 4A), in which many double substitutions have significantly lower DF compared to the double synonymous DF (Figure 4A). Combination NN contains only 4 cases with codon under positive selection and 4 cases compatible with purifying selection. Thus, neutrality cannot be rejected for the entire group (Figure 4A). The individual cases in combination NN that are compatible with positive selection are ACT → GTT (T → V), CCT → TTT (P → F), TCT → CTT (S → L), and TTT → CCT (F → P) (Supplementary Table S1).

## Discussion

Multiple mutations within the same codon have been claimed to be driven by positive selection (Bazykin et al., 2004; Rogozin et al., 2016; Belinky et al., 2018). This claim is consistent with the possibility that a prevalence of positively selected double nucleotide mutations is a compensation for the first deleterious mutation through subsequent positive selection (Bazykin et al., 2004; Rogozin et al., 2016; Belinky et al., 2018). The main goals of this work were to consider the mutational biases in the inference of selection in codon double substitutions and to understand whether codon double substitutions in yeasts and primates were under any type of selection compared to double synonymous substitutions. Just a few cases of elevated DF (<1 and 1% for human and yeast, accordingly) were detected for the combination NN. Such cases are compatible with previously reported positive selection on multiple nucleotide substitutions (Bazykin et al., 2004). Analysis of individual cases in primates and yeasts suggested that codons TTT (encoding phenylalanine) and CCT (encoding proline) are most frequent in terms of positively selected double substitutions (Supplementary Table S1).

Distributions of DF values for NS and SS double substitutions are not statistically different from NM1 and NM2 distributions (Figure 3), whereas SN and NN had significantly lower DF values suggesting that purifying selection substantially influences these classes of double substitutions in both primates and yeasts (Figure 3). In total, 15 and 34% double substitutions in primates and yeasts had significantly lower DF (after the Bonferroni correction), compatible with purifying selection. This result suggests that purifying selection affects a substantial fraction of double substitutions in codons. However, it is evident that in all four categories neutrality is the dominant mode of evolution (Figure 4).

We used synonymous sites as a control. Selection on synonymous sites have been previously shown in prokaryotes as well as in eukaryotes (Chamary and Hurst, 2005; Zhou et al., 2010; Gu et al., 2012; Lawrie et al., 2013; Shabalina et al., 2013; Long et al., 2018), while the reason behind this selection is not completely clear and could be contributed to stability of the DNA and staking effects (Goncearenco and Berezovsky, 2014), translational accuracy (Stoletzki and Eyre-Walker, 2007), and importance of secondary structure (Chamary and Hurst, 2005; Shabalina et al., 2013). Possible factors at the protein level are protein folding/structure (Oresic and Shalloway, 1998; Pechmann and Frydman, 2013) and a general selection at the amino acid level interacting with nucleotide replacements (Morton, 2001; Blazej et al., 2017). Although synonymous positions can be under some level of purifying selection, the same mutational forces are expected to influence codon non-synonymous double substitutions of the same bases, e.g., mutation rates that are influenced by specific bases would be similarly affected whether the mutation is synonymous or non-synonymous.

Previously, we assessed the selection that affects double substitutions within codons in prokaryotes (Belinky et al., 2019) using the same approach described in this paper. In many cases, it was found that codon double substitutions have significantly higher double/single ratios, compared to the same double synonymous substitutions (14%), suggesting that these are true cases of positive selection that acts on the second substitution and brings it to fixation in prokaryotes (Belinky et al., 2019). In primates and yeasts, we found just a few cases of putative positive selection (∼1%). Overall, the fraction of neutrally evolving codons is dramatically different: 11% in prokaryotes (Belinky et al., 2019) vs. 75% in primates and 65% in yeasts.

Recently it has been claimed that positive selection is overestimated by the branch-site test (BST), since most of the sites supporting positive selection are due to multinucleotide mutations (MNS) (Venkat et al., 2018). Phylogenetic tests of adaptive evolution, such as the widely used BST (branch-site test), assume that nucleotide substitutions occur independently. However, recent research has shown that errors at adjacent sites often occur during DNA repair/replication (Drake et al., 2005; Drake, 2007; Schrider et al., 2011; Stone et al., 2012; Terekhanova et al., 2013; Harris and Nielsen, 2014; Besenbacher et al., 2016), and the resulting MNS are overwhelmingly likely to be nonsynonymous (Venkat et al., 2018). Simulations under conditions derived from human and fly sequence alignments without positive selection show that realistic rates of MNS cause a systematic bias towards false inferences of selection (Venkat et al., 2018). This concern is certainly consistent with the observed substantial fraction of positively evolving double substitutions observed in prokaryotes (Belinky et al., 2019). However, the conclusion of the Venkat and co-workers (Venkat et al., 2018) requires a lot of caution, when applied to studied eukaryotes (primates and yeasts), where markedly fewer double substitutions are under positive selection (Figure 4).

The observed difference between pro- and eukaryotes (primates and yeasts) was observed previously for serine codons (Rogozin et al., 2016). Here, in the analyzed two eukaryotic lineages (yeast and primates), the difference of the DF of codon double substitutions over DF of the double synonymous in null models was much smaller than in prokaryotes (Belinky et al., 2019). This is consistent with the fundamental population-genetic theory (Lynch, 2007; Charlesworth, 2009; Loewe and Hill, 2010), whereby eukaryotes have substantially smaller effective population sizes than prokaryotes, and the consequent decrease in the power of selection most likely cause weaker pressure for restoration of amino acids that are under positive selection in prokaryotes, but not in studied eukaryotes (primates and yeasts). This hypothesis is also consistent with the observed larger fraction of positively and negatively selected double substitutions for yeasts compared to primates (Figure 4), which have much smaller population sizes.

The observed low fraction of deleterious intermediates associated with further positive selection (Figure 3) could be also due to various compensatory mechanisms at the RNA or protein level (Ellis, 1990; Fink, 1999; El-Brolosy and Stainier, 2017). For example, one reason for the higher complexity of eukaryotes compared to prokaryotes is the increased number of domain combinations found in eukaryotes, where, for example, binding domains have been added to existing catalytic proteins (Bjorklund et al., 2005). Thus, compensatory mechanisms at the level of interactions between proteins and domains within multidomain proteins are expected to be more abundant in eukaryotes compared to prokaryotes (Ekman et al., 2006; Bhaskara and Srinivasan, 2011). It should be noted that involvement of other non-trivial compensatory mechanisms in eukaryotes cannot be excluded. Future analyses of the impact of various compensatory mechanisms are likely to provide a clearer picture of eukaryote-specific trends of evolution.

## Data Availability

Publicly available datasets were analyzed in this study. This data can be found here: www.yeastgenome.org https://www.ensembl.org/index.html.
