# Supplementary figures and images for: No evidence for widespread positive selection on double substitutions within codons in primates and yeasts
Source: Front Genet. 2022 Sep 9;13:991249. doi: 10.3389/fgene.2022.991249 (PMC9500374; doi:10.3389/fgene.2022.991249)

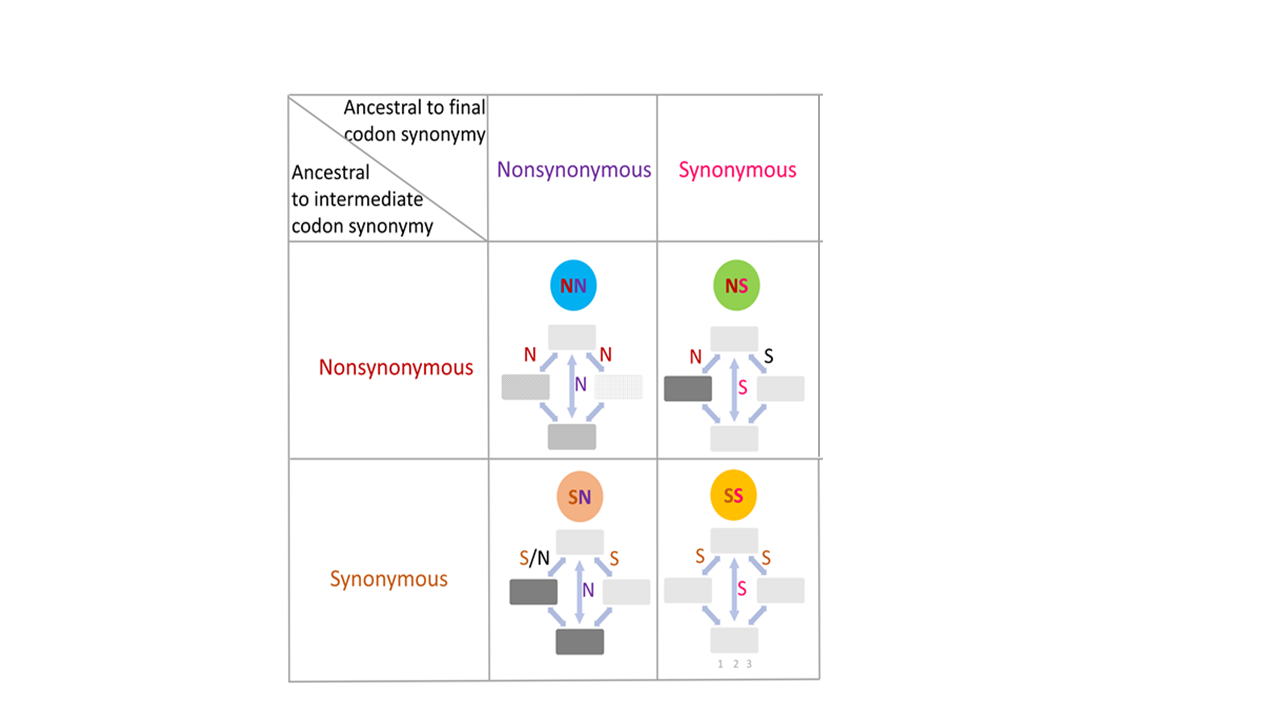

Supplement: Supplementary file 1 [file Image2.TIF]

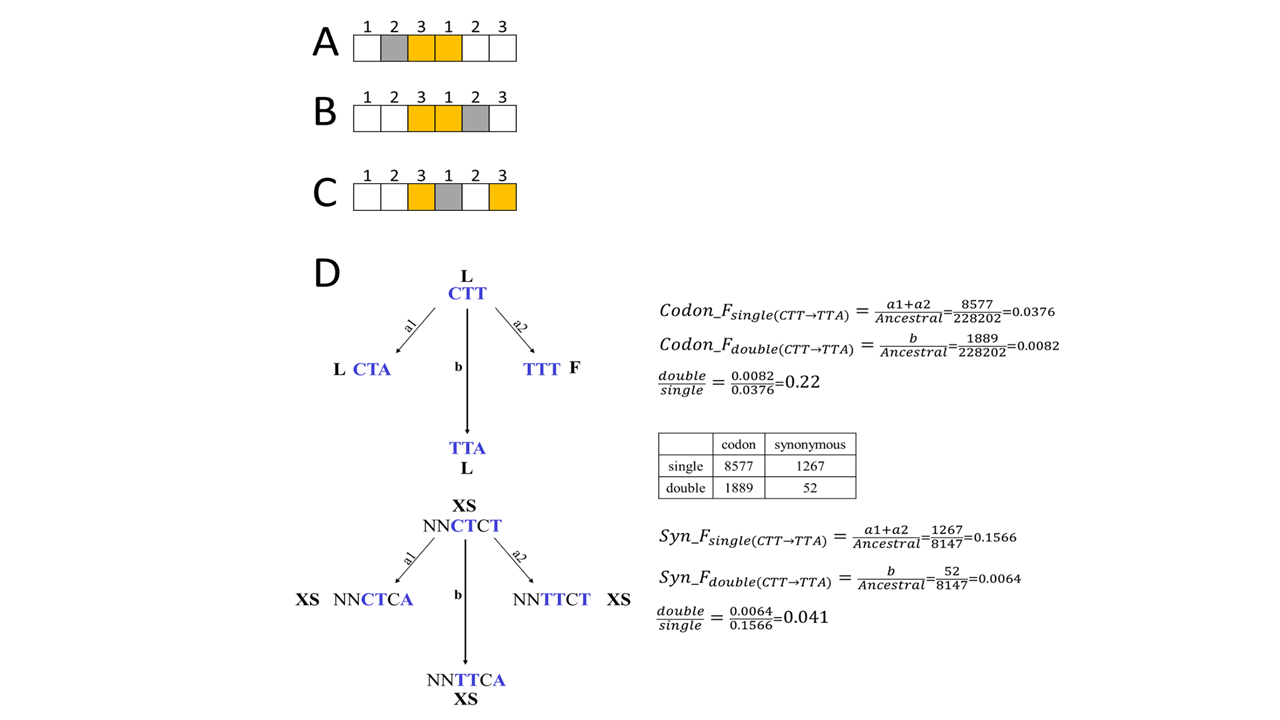

Supplement: Supplementary file 2 [file Image1.TIF]
